# Assessing the Fit of a Digitally Delivered National Diabetes Prevention Program Among Rural Living Adults: Qualitative Study

**DOI:** 10.2196/70406

**Published:** 2025-07-02

**Authors:** Gerit Wagner, Lyndsie M Koon, Patricia Smith, Kameron B Suire, Mary Hastert, Joseph E Donnelly, Melissa D Olfert, Paul Estabrooks, Anna M Gorczyca

**Affiliations:** 1 Division of Physical Activity and Weight Management Department of Internal Medicine University of Kansas Medical Center Kansas City, KS United States; 2 Research and Training Center on Independent Living University of Kansas Lawrence, KS United States; 3 CU Anschutz Health and Wellness Center University of Colorado Anschutz Medical Campus Aurora, CO United States; 4 Department of Kinesiology Berry College Rome, GA United States; 5 School of Agriculture and Food Systems West Virginia University Morgantown, WV United States; 6 Department of Health & Kinesiology College of Health University of Utah Salt Lake City, UT United States

**Keywords:** diabetes prevention program, rural, digital delivery, PRISM, qualitative, Practical, Robust Implementation and Sustainability Model

## Abstract

**Background:**

Rural living adults are disproportionately affected by type 2 diabetes compared to their urban counterparts. The Centers for Disease Control and Prevention’s National Diabetes Prevention Program (National DPP) is an evidence-based intervention that reduces the risk of type 2 diabetes through increased physical activity and modest weight loss, but overall reach remains limited, specifically in rural communities.

**Objective:**

This qualitative study aimed to examine the fit of the National DPP delivered digitally using Zoom or Facebook to rural living adults at risk for type 2 diabetes.

**Methods:**

Focus group scripts assessed the characteristics and perceptions of rural adults at risk for type 2 diabetes, infrastructure supports for implementation and sustainability, and external factors that could influence program fit. A reflexive thematic analysis was conducted separately on coded transcripts for each focus group. Themes were then deductively linked to the Practical, Robust Implementation and Sustainability Model domains.

**Results:**

Two focus groups were conducted with 14 participants after participating in the National DPP for 6 months, delivered through Zoom (n=9) or Facebook (n=5). Participants highlighted positive relationships between Practical, Robust Implementation and Sustainability Model constructs related to participant characteristics (ie, value of health improvements, weight loss, and reduced medication dependence as primary motivators) and perceptions of compatibility (ie, content alignment with participant needs) as well as infrastructure (ie, digital platforms provided better access) with program success in reach and engagement. Conversely, both formats were negatively impacted by interruptions in internet connectivity. External factors, such as referral pathways from local health care providers, could improve program reach. When considering differences between implementation infrastructure, Zoom facilitated greater social engagement and accountability compared to Facebook.

**Conclusions:**

This study identified contextual factors influencing the fit of digitally delivering the National DPP to rural living adults, including opportunities for using existing connections and health motivations to help improve acceptability, while tailoring curriculum, modality, and technology may improve appropriateness for rural populations.

**Trial Registration:**

ClinicalTrials.gov NCT05387434; https://clinicaltrials.gov/study/NCT05387434

## Introduction

Type 2 diabetes represents a significant public health concern, impacting the quality of life of those having the disease and accounting for US $412.9 billion (about US $1300 per person) through direct and indirect health care costs annually in the United States [1]. The prevalence of type 2 diabetes and prediabetes is 11.6% and 38% of the US population, respectively, and continues to rise [2,3]. To combat the increased prevalence of these conditions, in 2010, Congress authorized the Centers for Disease Control and Prevention (CDC) to implement the National Diabetes Prevention Program (National DPP), a large-scale, lower-cost version of the original 2002 Diabetes Prevention Program (DPP) [4]. The DPP is a behavioral weight loss program that emphasizes calorie reduction and increased physical activity (PA) to achieve a goal of 5%-7% weight loss over 6 months. The original DPP demonstrated a 58% decrease in the incidence of type 2 diabetes, as well as a 71% decrease in type 2 diabetes incidence in participants older than 60 years.

The reach of the National DPP, the largest translation of the original DPP, remains limited. As of 2022, there were only 2098 National DPP-recognized providers nationwide, and merely 3% of individuals diagnosed with prediabetes have participated in the program [5]. Substantial implementation barriers, including the inability to recruit high-risk populations coupled with the lack of sufficient financial reimbursement to build and sustain the program, have been reported [6]. Furthermore, access to and delivery of the National DPP is limited within communities where type 2 diabetes is more prevalent, including communities of lower socioeconomic status, rural counties [7], as well as communities of color, including Latinx and Indigenous communities, as well as older adults. Equal provision of programs such as the National DPP may mitigate rural or urban health disparities and address the disproportion in diabetes risk between different racial or ethnic and demographically located groups.

To address the limited reach of the program, the CDC currently recognizes delivery of the National DPP through several modalities including in-person, online (eg, asynchronous classroom with Lifestyle Coach interaction), distance learning (eg, telephone, Zoom [Zoom Video Communications], Skype [Microsoft Corp], FaceTime [Apple Inc], etc), or combinations of in-person, distance learning or online delivery [8]. Digital delivery of the National DPP, including online and distance learning platforms, eliminates the time and expense associated with lengthy travel to attend on-site programs, negating a significant barrier to participation for adults living in rural and challenging terrain [9]. Moreover, digital delivery of the National DPP may improve participation and retention of vulnerable populations [10,11], which are both important predictors of weight loss and reduced incidence of type 2 diabetes [6,12-15].

The US Cooperative Extension System (Extension) has been identified as a potential vehicle for the delivery of this program to rural communities [16]. Extension is a partnership between the US Department of Agriculture, land-grant universities, state, and local governments to improve research-based knowledge and application to communities. Extension uses nutrition and family consumer science agents in approximately 3200 offices across the United States as designated by the land-grant universities within each state. As of 2023, Extension delivered 20 National DPP programs across 16 states with a coordinated interest to expand access to the program [17]. Research indicates that distance learning delivery of the National DPP in rural areas by Extension agents is feasible and effective in achieving the program goals [18]. However, less is known about multilevel contextual barriers and facilitators of digital delivery of the National DPP in rural living adults. The Practical, Robust Implementation and Sustainability Model (PRISM) implementation framework was developed to improve the translation of evidence-based interventions into practice by identifying key contextual factors that are associated with program outcomes [19,20]. PRISM domains include (1) the intervention (organization and recipient perspectives), (2) the recipient (organizational and participant level characteristics), (3) implementation and sustainability infrastructure, and (4) external environment. The objective of this qualitative study was to use PRISM as the conceptual framework to examine the fit of the National DPP delivered digitally using Zoom or Facebook (Meta Platforms, Inc) to rural living adults at risk for type 2 diabetes.

## Methods

### Study Design

Focus groups were conducted in this cross-sectional qualitative study to gather information on a digitally delivered National DPP lifestyle change program for rural living residents by distance learning, where Extension personnel delivered live sessions over Zoom or online, asynchronously by a university research staff member via a private Facebook group (NCT 05387434). A comparison of intervention components in the pilot trial is provided in Table 1. Detailed methodology, feasibility, and effectiveness results of the pilot trial have been reported elsewhere [18]. Focus groups were chosen as the National DPP emphasizes group participation and social support [21]. Group dynamics encourage discussion and spontaneity that may not emerge in other methodologies, such as individual interviews. Further, focus group discussion helps highlight cultural norms across various groups that can help inform future research in rural-living adults with prediabetes [22].

**Table 1:** Overview of study components for a digitally delivered National Diabetes Prevention Program among rural living adults.

	Zoom	Facebook
Lifestyle coach	Extension agent	University research staff
Delivery modality	Zoom	Facebook
Curriculum	2016 PreventT2	2016 PreventT2
Contact	60-minute Zoom meeting	3 posts and direct messaging
Frequency	Weekly	Weekly
Duration	6 months	6 months
Self-monitoring device	Wearable and scale	Wearable and scale
Self-monitoring app	Fitbit	Fitbit

### Ethical Considerations

All participants provided informed consent to participate in the original study through REDCap (Research Electronic Data Capture; Vanderbilt University) electronic consent and were reconsented to participate in the focus group. This pilot study was approved by the University of Kansas Medical Center institutional review board and registered on ClinicalTrials.gov (NCT05387434). Study personnel implemented appropriate safeguards to prevent unauthorized use or disclosure of personal health information and implemented safeguards to protect the confidentiality, integrity, and availability of protected health information. Participants were pseudonymized through the transcription service to reveal only the gender identity or role of the speaker, that is, “F” for a female participant, “M” for a male participant, or “Q” for the interviewer. In the original pilot study, participants were compensated US $20 for time and travel to attend each of the 3 outcome testing visits (baseline, 3 months, and 6 months) and allowed to keep the Fitbit (Google LLC) and wireless scales used for self-monitoring in the pilot study. No compensation was provided for participation in the focus groups.

### Recruitment

In the original pilot study, members of the research team collaborated with participating Extension offices to recruit individuals via traditional media (ie, newspapers), social media, Extension-managed listservs, as well as distributing flyers at local businesses and physicians’ offices. Potential participants were directed to this study’s website, where they filled out an initial eligibility questionnaire. Once deemed preliminarily eligible, research staff met with potential participants via phone or Zoom to review and discuss the informed consent. Participation in the pilot study was required for participation in the focus group. Participants were offered the option to take part in focus groups about the acceptability and appropriateness of the National DPP at the end of the 6-month study via email. Purposive, convenient sampling was used with a minimum of 5 participants per focus group.

### Reflexivity and Positionality

A crucial aspect of performing a reflexive thematic analysis is to value the subjectivity and background of the researchers [23]. With regards to the research question, the senior author, AMG, has implemented lifestyle interventions in rural populations for the past 9 years [24,25]. AMG is an experienced scientist in lifestyle behavior interventions, and combined with this practical lived experience, while conducting both focus groups to facilitate participants’ identifying supports and barriers in this formative work. Research participants were informed of the experience of the senior researcher’s background, which helped encourage participant responses in the focus group data collection. This may have resulted in a level of translation that would be representative of other similar rural populations.

### Data Collection

**Figure 1:**
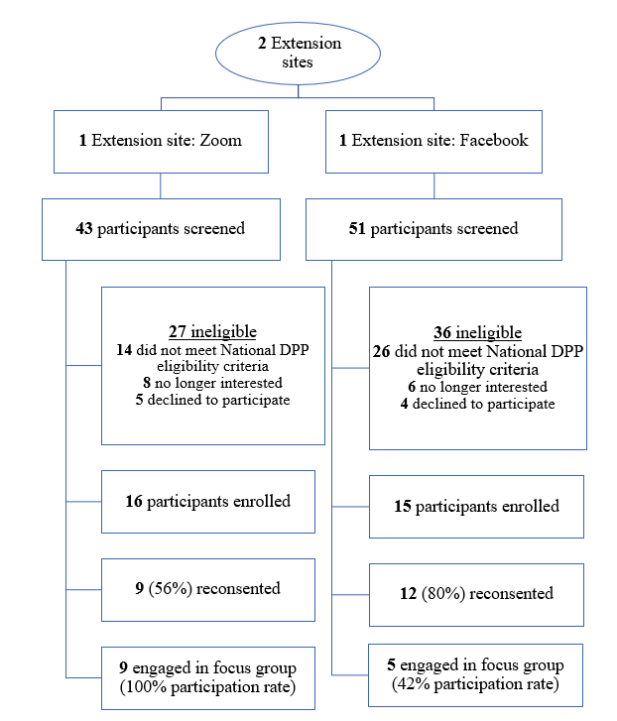
Flowchart of participants from the pilot study engaging in focus groups and included in the analysis, assessing the fit of a digitally delivered National Diabetes Prevention Program. DPP: Diabetes Prevention Program.

Baseline participant characteristics were assessed in March and May 2021. Study data were collected and managed using REDCap tools hosted at the University of Kansas Medical Center. All participants provided informed consent via REDCap. Focus groups were conducted by delivery modality (5-9 participants/group) via Zoom. Focus groups took place in August and October 2021. For Zoom participants, the focus group was held at a typical intervention meeting time to minimize scheduling conflicts. All Zoom participants who reconsented took part in the focus groups (9/9; Figure 1). As the Facebook group operated asynchronously without a set meeting time, the research team distributed a scheduling poll to determine availability and scheduled the Facebook focus group when the maximum number of participants were available. Of the 12 Facebook participants who reconsented, 5 (42%) participated in the focus groups. The focus group questions are presented in Textbox 1. Each focus group lasted approximately 60 minutes and was recorded and then transcribed verbatim by a third-party transcription service.

**Focus group guide to assess the fit of a digitally delivered national Diabetes Prevention Program among rural living adults.**

**Enrollment**

- Why did you participate in this program?
- What information specific to rural communities could have been included?
- How could the intervention or intervention materials have been tailored specifically for rural communities?

**Dietary changes**

- What types of dietary changes did you make?
- What helped you or could have helped you make dietary changes?
- What were some barriers to changing your diet?
- What would help you maintain your healthier eating habits?

**Physical activity**

- How did you get in the prescribed physical activity?
- Where did you do the physical activity?
- What types of activities did you do?
- What barriers did you encounter to increase your physical activity?

**Delivery modality**

- How did you feel about using Facebook as the primary platform for the program? (Facebook only)
- How did you feel about using Zoom as the primary platform for the program? (Zoom only)
- Did you have any issues with internet connectivity?
- Did you feel that you could connect with other group members virtually?

**Technology**

- Do you see yourself interacting with other group members using the Fitbit community option?
- Do you see yourself tracking your diet using a smartphone or iPad application?
- Tell me about your experience using the provided scale

**Other components**

- Would you like to tell us anything else about your experience with the Diabetes Prevention Program?

### Data Analysis Strategy

While the analysis was iterative in nature, it was conducted in six phases: (1) familiarization with the dataset; (2) coding; (3) generating initial themes; (4) developing and reviewing themes; (5) defining, refining, and naming themes; and (6) reporting [26]. For familiarization with the dataset, 3 researchers trained in qualitative analysis independently read through the focus group transcripts and familiarized themselves with participant responses, then constructed an initial codebook using MAXQDA software (VERBI Software, 2020). The codebook was refined through a second review to create a final codebook. Codes that addressed the primary aims were reviewed to develop themes. Themes, defined as patterns in the data that provide description and meaning to a qualitative dataset [27,28], were generated independently by the 3 initial researchers based on the primary aims of this study. Themes and subthemes were then discussed among all 3 researchers and were refined and defined until a consensus was met to align with PRISM domains. The COREQ (Consolidated Criteria for Reporting Qualitative Research) guidelines were followed (Multimedia Appendix 1) [29].

## Results

### Overview

Among the 31 participants enrolled in the pilot study (Zoom n=16; Facebook n=15), 14 (Zoom n=9; Facebook n=5) agreed to participate in the focus groups (Table 2). Notably, 93% (n=13) identified as female, 13% (n=2) identified as non-White and Hispanic or Latino, and 64% (n=9) possessed a college degree.

**Table 2:** Demographic characteristics of rural-living adults participating in focus groups to assess the fit of a digitally delivered National Diabetes Prevention Program.

		All pilot participants (n=31)	Focus group participants (n=14)	Zoom (n=9)	Facebook (n=5)
Age (years), mean (SD)		55.1 (12.8)	60.5 (9.3)	58.2 (10.6)	64.6 (3.8)
**Sex, n (%)**					
	Male	6 (19)	1 (7)	0 (0)	1 (20)
	Female	25 (81)	13 (93)	9 (100)	4 (80)
Racial or ethnic minority^a^, n (%)		5 (16)	2 (13)	2 (20)	0 (0)
BMI (kg/m^2^), mean (SD)		36.4 (7.8)	36.1 (9.4)	38.2 (5.4)	32.2 (13.1)
**Education, n (%)**					
	High school graduate	2 (7)	2 (14)	2 (22)	0 (0)
	Some college	5 (16)	3 (22)	2 (22)	1 (20)
	Bachelor’s degree	14 (45)	7 (50)	3 (33)	4 (80)
	Graduate degree	6 (19)	2 (14)	2 (22)	0 (0)
**Income (US $), n (%)**					
	<$40,000	6 (19)	4 (29)	3 (33)	1 (20)
	$40,000-$79,999	9 (29)	4 (29)	2 (22)	2 (40)
	>$80,000	15 (48)	6 (42)	4 (44)	2 (40)

^a^Non-White, Hispanic, Latino, other, or unknown ethnicity.

The finalized themes and subthemes were evaluated to describe the experience with the intervention for each group (Zoom and Facebook) and to compare between groups to provide a rich description of similarities and differences. Responses were categorized into four themes based on PRISM: (1) participant characteristics, (2) perceptions of program compatibility, (3) program delivery infrastructure, and (4) external factors influencing program success. The final set of themes and subthemes is presented in Table 3 with illustrative quotes.

**Table 3:** Themes and selected supporting quotes from rural living adults participating in focus groups to assess the fit of a digitally delivered National Diabetes Prevention Program.

I. Participant characteristics		
	Weight loss was a common goal	For me it was losing weight as a side which is a great one, but I do have several family members that have diabetes, and it caught my eye because by getting healthy, I can maybe possibly prevent diabetes. [Female, Zoom]
	Improve their laboratories or health numbers	I had been to the doctor and... my sugar was high and so she wanted to do an A1C check. And when she checked, my A1C was a little high. So she wasn't concerned, but I was. So I saw this advertised in the paper and decided that it was a way to be proactive about staying on top of that. [Female, Facebook]
	Desire to reduce or eliminate dependence on prescription medications	I was already on metformin in the morning and metformin at night when I got this. And now I'm down to one metformin at night and my blood sugar now is at – my A1c is 5.4. So I'm hoping that if I continue on this, I can get off of it completely. [Female, Zoom]
**II. Perceptions of program compatibility**		
	More information on how to eat healthier	I still think – I don't know. I was the one that said recipes were hard. The whole time, recipes were hard to find. You know?... So it'd be really cool if there was some kind of recipe book that went along with this, you know? [Female, Zoom]
	Challenges of integrating the program’s dietary recommendations into their daily lives	I think when I looked at how long it takes to do the food prep and then I look at what's available in the grocery store, I kind of understand why people just grab the plastic sack and open it. [Female, Facebook]
	Differences in accessibility and convenience of delivery modality	For me personally Zoom has been so nice because I can still take care of my family and there were nights that we had Girl Scout meetings and I didn't have to miss a meeting or – I could do it wherever, whenever. Right now I'm sitting outside with kids. [Female, Zoom]
**III. Program delivery infrastructure**		
	Recruited through a variety of mediums	I believe that I originally found the ad for it in the newspaper. [Female, Zoom]
	Health care providers could be better used in the recruitment process	Before I signed up for this, when I got the email, I went and talked to [my doctor]... I said, Have you ever heard about this? And he pointed and there was a flyer on his cabinet... And he goes, Is it this one? And I kind of looked and I said, Yeah, I'm pretty sure it is. And he goes, No because I've never had anybody go through that. And so I guess I'm thinking here I am somebody that is already on metformin. Why wouldn't you have promoted that with me? So I think you need to work with the doctors in rural areas more. [Female, Zoom]
	Differences in digital literacy impact self-monitoring behaviors	I'm not that good with the cell phone. [Female, Zoom]
	Digital self-monitoring apps increased participant accountability and motivation	Having to actually write down and record how many fruits and vegetables you eat is always eye-opening. I mean, I think I eat a lot of fruits and vegetables. But I eat more today than I did six months ago because I had to write them down and I had to account for each one. And if I eat more, then my family eats more too. [Female, Facebook]
	Use of self-monitoring apps in their daily lives	I used MyFitnessPal because I've used it before and I log – I have logged every single thing I've eaten for the last six months I guess, and it communicates with Fitbit. [Male, Facebook]
	Detailed knowledge of physical activity habits	And having the accountability of how many steps. Before, I used my phone. Well, if you don't take your phone everywhere, then you don't get all the steps. And so this is, again, a tool that can maybe make you more accountable. And I want to look into some of the other features that the Fitbit has that I haven't used like how well are you sleeping and some of that kind of stuff. [Female, Facebook]
	Social connections and accountability	Zoom: I did like the contact of seeing everybody. It kind of felt like you were more connected to everybody. [Female, Zoom] Facebook: Yes, I posted [to Facebook], but I don't think I felt there was a real connection. [Female, Facebook]
**IV. External factors influencing program success**		
	Various barriers to physical activity	I found excuses it was really hot. I just don't adjust to the heat as well anymore, and I know exercise helps that, but that and then we had mosquitoes this year like crazy, and that literally put on so much spray every day. [Female, Facebook]
	Strategies to overcome physical activity barriers	I got my bicycle in June and that's really been good for me mentally because I can just get on it and go by myself and just zone out. So that was good for me. And then I did get the thing to hook it to this winter so I can pedal and watch TV at the same time or read a book, whatever, so. [Female, Zoom]
	Technical challenges	Zoom: If [Lifestyle Coach] was at home and the wind and stuff, there was some internet issues that way. [Female, Zoom] Facebook: I don't know in rural areas using the technology platform. I know that the smaller the town, maybe the less comfortable they feel using it. Here in Salina, I thought it was great. But it depends on who we're trying to reach. I mean, I'm sure there's lots of people that the technology is not a turnoff or not a downside. [Female, Facebook]

### Participant Characteristics

#### Overview

Participants noted a variety of reasons for joining this study, characterizing many of their specific health needs and motives. Motives to participate in the program were similar across both groups and included weight loss, improving health metrics such as cholesterol and glycated hemoglobin (HbA_1c_), reducing medication dependence, and reducing the risk of disease progression (ie, type 2 diabetes).

#### Weight Loss Was a Common Goal Among Participants

Weight loss was often linked to their broader health objectives. Many participants recognized that losing weight could help them improve their overall health, reduce the risk of developing chronic diseases, and possibly eliminate the need for certain medications. They viewed the National DPP as an opportunity to achieve sustainable weight loss, which would in turn lead to better health outcomes.

#### Participants Wanted to Improve Their Laboratory or Health Numbers

Many participants were motivated to enroll in the program to improve their health metrics, particularly laboratory results such as HbA_1c_ levels and cholesterol. They expressed concern about their current health status and wanted to take proactive steps to avoid more serious health issues, such as a diabetes diagnosis. Participants mentioned how their doctors had warned them about rising HbA_1c_ levels, prompting them to join the program to better manage their blood glucose.

#### Participants Were Driven by the Desire to Reduce or Eliminate Their Dependence on Prescription Medications

Some were already taking medication for conditions such as high blood pressure or diabetes, and hoped that by participating in the program, they could lower their dosage or stop taking the medication altogether. This goal of reducing medication use was a significant motivator for many, as they viewed it as a marker of improved health and well-being.

### Perceptions of Program Compatibility

#### Overview

Participants discussed how the content and delivery modalities of the program aligned with the needs of rural living adults at risk for diabetes, including how the content of CDC’s Prevent T2 curriculum met their stated needs and opportunities to further strengthen program content for future interventions. Participants from both groups found the curriculum valuable but desired more structure and guidance with topics such as recipes and meal planning. Further, participants noted differences in how the different delivery modalities accommodated their schedules, lifestyles, and social needs.

#### Participants Desired More Information on How to Eat Healthier

While the program provided some guidance, participants felt that more detailed and structured information would have been beneficial. This included more recipes, guidance on managing portion sizes, and strategies for incorporating healthier foods into their daily routines. Some participants expressed the need for more practical resources, such as a recipe book or meal planning guides, to help them incorporate more fruits and vegetables into their diets. The lack of clarity around certain dietary concepts, such as understanding carbohydrates, was also highlighted as a barrier to making informed food choices.

#### Participants Expressed Challenges Integrating the Dietary Recommendations Into Their Daily Lives

Many found it difficult to make healthier food choices consistently, especially when faced with limited time or access to fresh ingredients. The shift toward fresh produce and away from processed foods was a significant change for many, requiring a considerable adjustment in shopping and meal preparation, and budget. Participants also noted that dietary guidance was sometimes at odds with family needs. One challenge mentioned was the responsibility as the primary household food preparer, needing to satisfy the dietary needs and preferences of family members. Others noted the inconvenience of preparing healthy foods as a challenge in long-term adherence to dietary changes. Furthermore, participants highlighted how eating out and social gatherings conflicted with the program’s recommendations, as participants found it difficult to maintain their healthy eating habits in these settings.

#### Participants Noted Differences in Accessibility and Convenience of Delivery Modality

##### Zoom

Participants generally found Zoom a highly convenient platform for participating in the program. It allowed them to join sessions from home, making it easier to integrate the meetings into their daily schedules. The ability to participate from anywhere, such as while caring for children or handling other responsibilities, was particularly appreciated. However, a few participants mentioned occasional internet connectivity issues that sometimes disrupted the experience.

##### Facebook

Participants generally found the Facebook group flexible and convenient, allowing them to engage with the program at their own pace. The ability to access materials and participate in discussions asynchronously was appreciated, particularly by those with busy schedules. However, some participants missed the immediacy of real-time interaction, which they expressed would have enhanced their engagement and accountability.

### Program Delivery Infrastructure

#### Overview

Participants discussed their experiences with the program’s infrastructure, including perceptions of the recruitment methods used and opportunities for improving the reach of recruitment efforts. Further, participants discussed how the technological infrastructure, including provided hardware and software apps, influenced their health behaviors and social interactions, as well as how this same technology presented unintended barriers to their participation in the program.

#### Participants Were Recruited Through a Variety of Media

Participants were recruited for the program through various channels, including newspaper advertisements, newsletters from local Extension services, word of mouth, and emails. Some participants mentioned discovering the program by chance, such as reading about it in a local paper or hearing about it from a neighbor. Despite the variety of recruitment methods, some felt the outreach could have been more extensive to reach a wider audience.

#### Health Care Providers Could Be Better Used in the Recruitment Process

Some participants expressed frustration that health care providers were not as involved in the recruitment process as they could have been. Several participants noted that although they discussed their health issues, such as rising HbA_1c_ levels, with their doctors, the program was not recommended to them directly. In some cases, participants found out about the program independently and mentioned it to their doctors afterward, who were then supportive but had not been proactive in suggesting the program. The consensus was that health care providers could play a more active role in promoting such programs, given their professional authority and influence on patients’ health decisions.

#### Differences in Digital Literacy Impacted Self-Monitoring Behaviors

Some participants expressed confusion about using the apps for intended self-monitoring purposes, while others mentioned how they could tailor the apps to track specific activities or set personalized goals, including step counts and protein or calorie goals, making the tools more relevant to their unique health goals.

#### Digital Self-Monitoring Apps Increased Participant Accountability and Motivation

Participants mentioned the Fitbit and MyFitnessPal apps improved their ability to track steps, monitor progress, and receive reminders that kept them motivated to meet their daily PA goals. The real-time feedback provided by these tools acted as a constant motivator, encouraging participants to stay active and adhere to their exercise routines.

#### Participants Discussed the Use of Self-Monitoring Apps in Their Daily Lives

Many participants appreciated how straightforward it was to log their activities and monitor their progress. The synchronization between the Fitbit wearable device, the Fitbit smartphone app, and MyFitnessPal was particularly valued, as it allowed participants to track both PA and dietary intake in 1 place, simplifying the self-monitoring process.

#### Participants Valued the Detailed Insights Apps Provided Into Their PA

Features such as step counting, heart rate monitoring, and calorie expenditure allowed participants to gain a deeper understanding of their personal behaviors. This detailed tracking helped participants set more specific goals and measure their progress over time, which was crucial for maintaining their commitment to the program.

#### Social Connections and Accountability Felt Different Among Participants

##### Zoom

The visual and real-time interaction on Zoom significantly contributed to a sense of engagement among participants. The requirement to join live sessions and interact with others encouraged participants to stay on track with the program. Many noted that seeing each other’s faces and hearing voices fostered a supportive environment. The structured environment of scheduled meetings also provided a sense of discipline, which many participants found beneficial for their progress in the program.

##### Facebook

Engagement in the Facebook group varied, with some participants actively contributing while others were more passive by observing. The lack of real-time meetings or structured schedules made it easier for some participants to disengage. Nonetheless, those who did participate valued the ongoing support and found the platform to be a useful tool for maintaining accountability, especially when paired with tools such as Fitbit for tracking PA. However, the absence of regular, live interaction was seen as a barrier to sustained engagement and accountability for some. Some participants expressed discomfort with posting publicly within the private Facebook group, leading to less frequent and less personal interactions. Others noted that while the group allowed for some level of connection, it did not foster the same sense of community and support that could have been achieved through in-person or distance learning modalities.

### External Factors Influencing Program Success

#### Overview

Participants discussed how external influences shaped their ability to successfully engage with the program. Environmental barriers, modality-specific challenges, and other logistical constraints created barriers to sustained behavior change.

#### Various Barriers to PA Were Identified

Barriers included environmental factors such as heat, pests such as mosquitoes, and limited access to exercise facilities. Some participants mentioned that they did not feel comfortable exercising in public places such as gyms, while others found it difficult to maintain a regular exercise routine due to busy schedules or physical limitations, such as recovering from surgery. These barriers often hindered participants’ ability to engage in consistent PA.

#### Participants Strategized to Overcome External PA Barriers

Many adapted their routines to fit their circumstances, such as walking indoors during hot weather or using home exercise equipment. Some participants took creative approaches, such as walking in circles around their house or using local parks and tracks for outdoor exercise.

#### Different Technical Challenges Experienced by Delivery Modality

##### Zoom

While participants generally adapted well to the Zoom platform, a few did experience technical challenges, primarily related to internet connectivity. These issues were often minor and were typically resolved quickly, allowing participants to continue engaging with the program. Despite these occasional technical difficulties, Zoom was seen as a manageable and effective platform for delivering the intervention.

##### Facebook

Minimal technical challenges were reported in using Facebook for the program. Most participants found the platform familiar and easy to navigate. However, the asynchronous nature of the interaction sometimes led to delayed responses and a less dynamic exchange of ideas, which some participants found limiting.

## Discussion

### Principal Findings

This study provides important insights into the appropriateness of program recruitment, curriculum, digital delivery, and technology usage for self-monitoring in the context of the National DPP. For acceptability, rural living adults thought rural health care providers could play a larger role in program awareness and referrals. For appropriateness, there was a desire for more structured nutrition digital resources in the curriculum. Distance learning using Zoom fostered group communication and accountability, while online delivery using Facebook hindered participant social interaction. Self-monitoring weight and PA using wireless scales and wearable fitness devices helped reinforce behavior change and increase accountability to the program goals. Overall, results indicate considerations for the implementation of the National DPP using digital delivery strategies for rural living adults.

Participants reported that acceptability may be improved through greater health care providers’ awareness of the National DPP to enable a referral process for patients at risk for type 2 diabetes. Low rates of health care provider referrals have been a longstanding barrier to implementation of the National DPP, which is even more detrimental in rural communities [30]. Active recruitment strategies, which use collaborations with the medical community for participant referral, have the potential to improve National DPP recruitment [8] beyond that of traditional passive recruitment, which relies on potential participants to initiate contact with the National DPP. Traditional passive recruitment methods often lead to a population comprised predominantly of highly educated women [31] and lacking racial diversity [32]. Leveraging active recruitment methods, including point-of-care or population health management, may lead to greater participation in general and that of underrepresented populations in diabetes prevention trials [33]. Previous research has found that providers using the electronic health record were more likely to refer to the National DPP, which may provide a starting point for community organizations initiating conversations with clinical entities for recruitment [34]. Further, participants did not note that the program being delivered by an Extension agent negatively impacted program acceptability, suggesting that program quality remains consistent when delivered by Extension. However, the absence of explicit questions regarding the impact of the facilitating group’s lifestyle coach may limit the strength of this conclusion.

Despite the National DPP’s overall focus on diet quality, participants expressed a desire for structured guidance surrounding nutrition-related changes. The 2021 PreventT2 curriculum underwent updates emphasizing “whole foods and healthy eating patterns,” promoting “greater flexibility in allowing participants to tailor approaches to healthy eating and weight loss support” [35]. These updates align with professional consensus advocating for the individualization of eating plans and patterns to enhance adherence to healthy behaviors [36]. Despite the focus on overall dietary quality over specific benchmarks, focus group participants voiced a need for additional guidance and specific resources to facilitate dietary and food selection changes. However, the National DPP does not mandate that lifestyle coaches possess nutrition credentials, limiting their capacity to offer evidence-based clarifications or resources to support these changes. Recent evidence suggests the PreventT2 curriculum does not support meaningful changes in diet quality [37]. This presents a dilemma: participants seek more structured information, while the professional consensus suggests that rigid guidelines are ineffective in fostering long-term behavior change, a conflict difficult to resolve within the National DPP framework [36,38].

Our results suggest that differences in effectiveness between distance learning and online may, in part, be due to increased social engagement and accountability with the distance learning modality. A recent analysis of all National DPP participants through December 31, 2019, found greater weight loss for distance learning (4.7%) compared to online (2.6%) [39]. However, online, asynchronous delivery of the National DPP may be associated with lower costs than other modalities [40]. Using digital delivery, including both distance learning and online modes, may overcome barriers specific to rural communities in accessing effective lifestyle interventions, such as the lower availability of programs, transportation, and other caretaking duties. Organizations should weigh the increased favorability and higher rate of weight loss associated with distance learning delivery against the convenience and accessibility that online delivery can provide.

There was high acceptability in using the wireless scale and wearable fitness tracker to self-monitor, which helped participants’ accountability and motivation to engage in PA and monitor body weight. Self-monitoring weight and PA are cornerstone health behaviors targeted in the National DPP. Wearable devices may empower participants to engage in PA; however, the efficacy of the device alone as a behavioral strategy is unknown [41,42]. This study provided both the scale and fitness trackers to participants, which may not be financially appropriate for all programs. Furthermore, there are sociodemographic disparities in wearable ownership that skew toward younger, educated adults living in urban areas [43]. Stakeholders interested in increasing the uptake of their National DPP should consider the provision of scales and fitness trackers, as rural adults in this study felt they were helpful.

The results of this study align with results from various demographic groups within the United States as well as DPPs present in other countries. In the United States, rural communities can exhibit substantial heterogeneity. This diversity in rural-living adults presents itself as a barrier in designing culturally appropriate materials for rural communities, as needs may differ with geographic location and sociodemographic characteristics [44]. However, the sentiments expressed by the participants in this study have been echoed across diverse demographic groups’ participation in the National DPP as well as other DPPs internationally. Interventions conducted in California note the positive impact virtual platforms may offer in terms of increasing program reach [45,46]; however, concerns regarding the potentially negative impact of digital platforms on participant engagement and peer support remain. Additionally, a faith-based community intervention among African American participants noted the importance of self-monitoring through weight tracking and similar desires for more in-depth program materials, similar to the interest in expanded nutrition resources observed in our study [47]. Internationally, the effectiveness of DPPs is well documented [48], and a recent systematic review including studies from the United States, England, and Australia examining health care workers’ perspectives on barriers and facilitators for referrals to DPPs matched the sentiments of participants in our study: there is a need for DPPs to further strengthen the role of health care providers in referring participants to these programs [49]. Literature regarding perceptions of individuals participating in digital DPPs, specifically, however, is less abundant. International digital DPPs, such as the Irish National Diabetes Prevention Programme and the National Health Service Healthier You*:* Digital Diabetes Prevention Programme in England, note findings similar to those noted within our study [50,51]. Participants in both programs have highlighted the role of health care providers in recruiting participants and have emphasized the need for greater provider engagement to encourage participation and program uptake. Further, participants acknowledged that even in digital settings, personalized support from lifestyle coaches was important for accountability [52]. Self-monitoring tools, such as tracking food intake, were also noted to be of importance for some participants. These findings underscore the shared challenges and opportunities in diabetes prevention efforts across diverse populations and settings.

### Limitations

While our study provides important insights on the acceptability and appropriateness of digital delivery of the National DPP to rural adults, it is not without limitations. Given the increasing diversity of rural populations, this study may not reflect the experiences of other geographic areas due to the relative sample homogeneity and small sample (n=14). Second, the planned duration of the feasibility study was originally only 6 months. While the National DPP is traditionally a 12-month program, this study may not represent the perspective of individuals who experience the full year-long program. However, as the National DPP requires at least 16 sessions conducted within the first 6 months of the program and 6 sessions in the final 6 months, participants were exposed to most of the curriculum at the time of the focus groups [8]. Finally, conducting focus groups required participants to reconsent to the research process, which resulted in a relatively low response rate in the Facebook group. Therefore, results do not fully represent all online delivery modalities of the National DPP and are specific to Facebook.

Despite these limitations, this study fills an important gap in the perception of digital delivery to improve the reach of the National DPP to rural adults using an evidence-based implementation framework (PRISM). Furthermore, the use of a reflexive thematic analysis enhanced the ability to analyze findings within and between groups, specifically the acceptability of distance learning and online delivery modalities. Leveraging the perspectives of rural living adults provides robust information that can be used for developing and testing future implementation strategies to improve program accessibility.

### Future Directions

To enhance the reach and impact of the National DPP, future research should explore ways to improve recruitment strategies, particularly by engaging health care providers in rural and underserved communities to actively refer high-risk individuals. Furthermore, integrating culturally tailored dietary and PA resources could improve the program’s acceptability across diverse populations. Additionally, the effectiveness of emerging digital self-monitoring tools, such as wearable devices, should be assessed for their long-term impact on health outcomes and participant adherence, particularly in low-resource settings. Finally, comparative research evaluating cost-effectiveness between different delivery modalities (eg, distance learning versus online) is essential to inform scalable, sustainable interventions that reduce health disparities in rural areas.

### Conclusions

This study highlights the potential of digital delivery, through both distance learning and online modalities, to expand the reach of the National DPP to rural populations. While digital modalities offer significant advantages in accessibility and flexibility, differences in participant engagement, social support, and technology use across platforms must be addressed to enhance program effectiveness. The results suggest that tailoring recruitment strategies, particularly through health care provider involvement, and offering more structured dietary guidance could improve program acceptability and outcomes. Ultimately, these insights underscore the need for continued research to optimize digital delivery approaches and ensure equitable access to diabetes prevention resources in underserved communities.

## Appendix

Multimedia Appendix 1 COREQ checklist.

## Abbreviations

CDC: Centers for Disease Control and Prevention
COREQ: Consolidated Criteria for Reporting Qualitative Research
DPP: Diabetes Prevention Program
Extension: US Cooperative Extension System
HbA1c: glycated hemoglobin
National DPP: National Diabetes Prevention Program
PA: physical activity
PRISM: Practical, Robust Implementation and Sustainability Model
REDCap: Research Electronic Data Capture
